# Optimization of the production of knock-in alleles by CRISPR/Cas9 microinjection into the mouse zygote

**DOI:** 10.1038/srep42661

**Published:** 2017-02-17

**Authors:** Aurélien Raveux, Sandrine Vandormael-Pournin, Michel Cohen-Tannoudji

**Affiliations:** 1 Institut Pasteur, CNRS, Unité de Génétique Fonctionnelle de la Souris, UMR 3738, Department of Developmental & Stem Cell Biology, 25 rue du docteur Roux, F-75015 Paris

## Abstract

Microinjection of the CRISPR/Cas9 system in zygotes is an efficient and comparatively fast method to generate genetically modified mice. So far, only few knock-in mice have been generated using this approach, and because no systematic study has been performed, parameters controlling the efficacy of CRISPR/Cas9-mediated targeted insertion are not fully established. Here, we evaluated the effect of several parameters on knock-in efficiency changing only one variable at a time. We found that knock-in efficiency was dependent on injected Cas9 mRNA and single-guide RNA concentrations and that cytoplasmic injection resulted in more genotypic complexity compared to pronuclear injection. Our results also indicated that injection into the pronucleus compared to the cytoplasm is preferable to generate knock-in alleles with an oligonucleotide or a circular plasmid. Finally, we showed that Cas9D10A nickase variant was less efficient than wild-type Cas9 for generating knock-in alleles and caused a higher rate of mosaicism. Thus, our study provides valuable information that will help to improve the future production of precise genetic modifications in mice.

Genetically modified animals are critical for understanding the mechanisms of biological processes and the physiopathology of human diseases. In particular, the ability to generate knock-in (KI) mutations allows both efficient analysis of gene expression and precise dissection of gene functions. In the mouse, deliberate and controlled genetic modifications were, until recently, mostly achieved through homologous recombination in embryonic stem (ES) cells, a powerful but laborious and time-consuming process^1^,^2^. Following pioneer works with yeast I-SceI nuclease^3^,^4^, the development of methodologies allowing the generation of targeted double strand breaks (DSBs) in zygotes using endonucleases such as Zinc finger nucleases or transcription activator-like effector nucleases has greatly accelerated the production of genetically modified mice, rats and other organisms^5^. Indeed, DSBs locally stimulate two major repair pathways. First, the non-homologous end joining (NHEJ) pathway, an error-prone mechanism that introduces small insertions or deletions (indels) at the repair junction and usually causes gene disruption. The second is the homology-directed repair (HDR) pathway, which uses a DNA donor template sharing homology with the sequences flanking the break for precise repair, leading to the production of animals with KI alleles. More recently, the advent of CRISPR/Cas systems has taken mutagenesis one step further, as Cas9 nuclease can be directed by short RNAs to induce, with high efficiency, a DSB at a precise genomic location^6^,^7^. Today, CRISPR/Cas9 genome editing in early embryos is becoming the privileged approach to perform programmed genetic modifications in a wide variety of species^8^,^9^.

The first CRISPR/Cas9-edited mouse lines were obtained using direct injection of *Streptococcus pyogenes* Cas9 mRNA and synthetic single guide RNAs (sgRNAs) together with or without a repair matrix into one-cell embryos^10^,^11^,^12^. Since, genome editing at various loci has been reported using a similar procedure^13^,^14^,^15^,^16^,^17^ or modified protocols to avoid the delicate step of embryo micromanipulation^18^,^19^,^20^, enhance the production of edited alleles^21^,^22^,^23^ or generate more complex alleles^24^,^25^. Small deletions and non-functional alleles resulting from the NHEJ repair pathway were obtained with high frequency (usually 50–100% of the embryos/pups)^12^,^26^. A smaller number of KI alleles have been generated through the HDR pathway and reported targeting frequencies were much lower (usually 1–20%)^13^,^17^. Because many factors can influence the experiment outcome and no systematic study has been performed, the parameters controlling the efficacy of KI and their relative contributions are not fully established. Here, we investigated the role of several parameters on the generation of KI alleles by comparing conditions differing from each other by only one parameter.

## Results

### Experimental design

To determine the parameters potentially controlling CRISPR/Cas9-mediated KI efficiency following microinjection of sgRNA, Cas9 messenger RNA and a repair matrix into the mouse zygote, we performed a systematic analysis changing only one variable at a time. The parameters we tested are the concentrations of microinjected compounds, the intracellular site of injection, the nature of the repair matrix and the use of Cas9 nickase (Cas9n) variant. Double nicking by Cas9n and two paired sgRNAs targeting sequences 30 to 70 bp apart from one another and located on opposite strands allows to enhance genome editing specificity by reducing off-target effects^27^,^28^. The effects of other parameters, such as the genotype of the zygotes or the targeted locus, were not addressed in our study and to minimize their impact, all experiments were performed on a unique genomic location, using zygotes of the same genotype that were microinjected by a single experimenter. After microinjection, embryos were cultured up to embryonic stages ranging from 8-cell to early blastocyst and assessed for genomic modifications at the targeted region by PCR amplification. This allowed the rapid identification of HDR events as well as nonspecific indels consecutive to DSB repair by NHEJ. Mosaic embryos harbouring 3 or more alleles were also readily identified. Most conditions were repeated at least twice in order to balance experimental variations and strengthen our conclusions.

**Figure 1:**
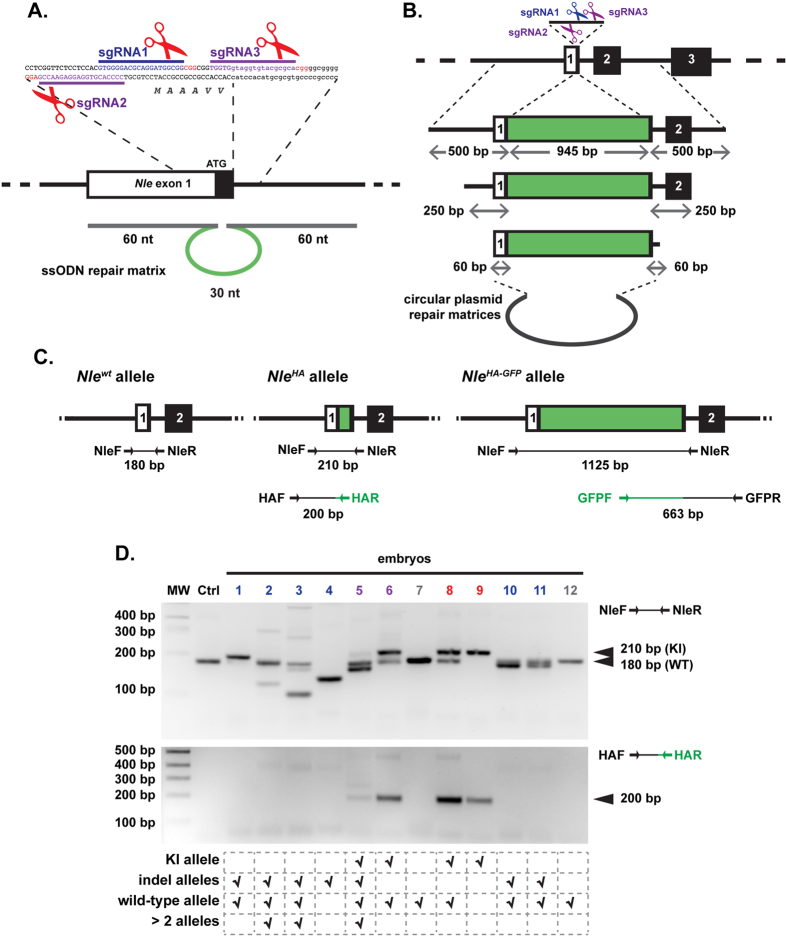
CRISPR/Cas9 genome editing strategy and mutation detection at the *Nle* exon 1 region. (**A**) Schematic representation of the *Nle* exon 1 region, sgRNAs sequences and ssODN repair matrix. The coding part of *Nle* exon 1 is indicated in black. The sgRNA-targeting sequences are underlined, and the protospacer adjacent motif sequences are labelled in red. Cas9 nuclease cuts DNA at position −3 bp from the protospacer adjacent motif. The ssODN repair matrix contains 60 nucleotides of homology flanking both sides of the DSB generated by sgRNA1. (**B**) Schematic representation of the dsDNA matrices used in this study. (**C**) Schematic representation of *Nle*^*wt*^, *Nle*^*HA*^ and *Nle*^*HA-GFP*^ alleles. Positions of the primers and sizes of the PCR fragments are indicated below each allele. (**D**) Agarose gel electrophoresis analysis of PCR products of one non-injected embryo (Ctrl) and twelve embryos (lanes 1 to 12) microinjected into the pronucleus with Cas9 mRNA/sgRNA1/ssODN (100 ng/μl/50 ng/μl/20 ng/μl) and cultured for 72 hours. The interpretation of the PCR profiles is given below the gel. Embryo n° 12 has no edited allele and was considered wild-type. Some embryos displayed more than 2 alleles and were considered mosaic (n° 2, 3 and 5). Only the KI allele was detected in embryo n° 9 which was considered homozygous for the KI allele. MW: 100 bp molecular weight marker. Embryos with no mutations, indels, KI allele and both KI allele and indels are labelled in gray, blue, red and purple respectively.

Genome editing was targeted to the first exon of the *Notchless (Nle*) gene at the vicinity of the ATG. To introduce a tag at the amino-terminal extremity of the NLE protein, we designed a CRISPR/Cas9-based strategy to induce DSBs either with the wild-type Cas9 enzyme and the sgRNA1 guide sequence or with the Cas9D10A nickase variant and sgRNA2 and sgRNA3 paired RNA guides (Fig. 1A). Two types of repair matrices were used: a single stranded oligo DNA nucleotide (ssODN) comprising 30 nt coding for the small HA tag flanked by a 60 nt homology arm on each side (Fig. 1A), and a circular double stranded plasmid containing a 945 bp region coding for in frame HA and GFP tags flanked by 500 bp homology regions (Fig. 1B). Following HDR, the expected *Nle*^*HA*^ and *Nle*^*HA-GFP*^ knock-in alleles are predicted to be refractory to re-editing with the wild-type Cas9 strategy, because the complementary DNA sequence to sgRNA1 is interrupted by the insertion of the tag sequences. The same is true for the Cas9n strategy, since the complementary DNA sequences to sgRNA2 and sgRNA3 lie too far from each other after HDR (90 bp and 1007 bp respectively) for efficient DSBs generation by double nicking^27^,^28^. Primers used to evaluate indels and KI frequencies are shown on Fig. 1C and Supplementary Table S3. An illustration of mutations analysis on microinjected embryos is given on Fig. 1D. Experiments were performed using embryos with a mixed genetic background, a proportion of which harbouring single nucleotide polymorphisms within the region covered by the repair matrices (Supplementary Fig. S1). Since HDR can be inhibited by small heterologies between the targeted locus and the repair matrix^29^,^30^, KI frequencies determined in this study are likely lower than those that would be obtained in a fully isogenic situation.

### KI efficiency with ssODN repair matrix depends on Cas9 mRNA/sgRNA concentration but not on the site of injection

As a starting point, we chose to perform pronuclear injection using RNAs concentrations (5 ng/μl Cas9 mRNA, 2.5 ng/μl sgRNA1) defined in the seminal work of Yang and collaborators^12^ and three concentrations of the ssODN repair matrix (2, 20 and 40 ng/μl). We observed a marked increase in KI efficiency (5% vs 15%) when ssODN concentration was increased from 2 to 20 ng/μl (Table 1). KI efficiency then dropped down when concentration was further increased to 40 ng/μl (3%, Table 1). We therefore decided to perform our study using the intermediate ssODN concentration.

**Figure 2:**
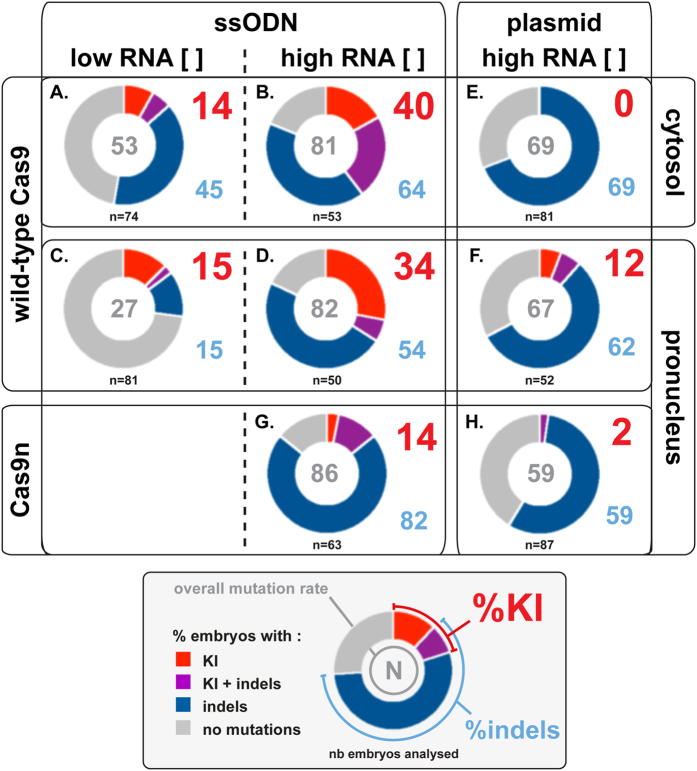
Comparison of the effect of several parameters on KI efficiency. Each circle represents the proportion of embryos with KI, indels, KI and indels, or wild-type only alleles for a given condition. The overall mutation rate, which corresponds to the proportion of embryos displaying at least one edited allele, is indicated at the center of each disk. Because some embryos contained both KI and indels alleles, the overall mutation rate can be less than the sum of the rates of KI and indels alleles. High concentration ([]) correspond to 100 ng/μl of Cas9 mRNA and 50 ng/μl of sgRNA while low [] corresponds to 5 ng/μl of Cas9 mRNA and 2.5 ng/μl of sgRNA. ssODN and circular plasmid were injected at 20 and 40 ng/μl respectively.

We first asked whether cytosolic or pronuclear microinjection led to different KI efficiencies. Indeed, Cas9 mRNA has to pass through the cytoplasm to be translated while the ssODN is required in the nucleus for the HDR process. The compartment in which sgRNA is being loaded on Cas9 protein has not been characterized yet. Therefore, whatever the site of injection, conditions are predicted suboptimal for at least one of the components that will need to diffuse from one compartment to the other. We performed cytosolic and pronuclear microinjection with either low (5/2.5 ng/μl) or high (100/50 ng/μl) Cas9 mRNA/sgRNA concentrations while keeping the concentration of ssODN repair matrix constant (20 ng/μl) (Table 1, Fig. 2A–D). We found that, for a given RNA concentration, KI efficiency was identical whatever the site of injection (compare Fig. 2A with 2 C and 2B with 2D), suggesting that the ssODN was able to rapidly diffuse to the nucleus when injected in the cytoplasm. Strikingly, KI frequency increased significantly when more Cas9 mRNA and sgRNA were delivered into zygotes (40% vs 14%, p < 0.001 and 34% vs 15%, p < 0.01 for cytoplasmic and pronuclear injection respectively), consistent with the expectation that more DSBs are induced under high RNA concentration condition. Similar results were obtained when genome editing was targeted to the *Sox2* locus (Supplementary Fig. S2 and Table S1).

Interestingly, with the low but not the high RNA concentration, the rate of indels was higher for cytoplasmic compared to pronuclear injections (45% vs 15%, p < 0.0001). A possible explanation could be that pronuclear injections with low RNA concentrations resulted in a delayed timing for efficient DSBs induction. Prominence of NHEJ and HDR repair pathways has been shown, in cellular models, to vary during the cell cycle, with HDR being more prevalent in S and G2 phases (reviewed in ref. 31). If this is also true for the mouse zygote, and given the fact that injections were performed in G1/early-S phase (20–24 h post hCG^32^), postponing DSB induction towards the late-S/G2 phase may impinge on the rate of indels without affecting KI efficiency.

### Pronuclear injection improves KI efficiency with a dsDNA repair matrix

We next monitored KI efficiency with pronuclear injection of a circular double stranded plasmid. We first tested three concentrations of plasmid (4, 40 and 80 ng/μl) using pronuclear injection with high concentrations of Cas9 mRNA/sgRNA1 (Table 1). At 40 ng/μl, HDR events were easily detected while no or fewer KI events were observed with 4 and 80 ng/μl. We therefore compared the effect of pronuclear versus cytoplasmic injection using 40 ng/μl of plasmid. Of note, this corresponds to 1.0 × 10^10^ molecules/μl, which is more than one order of magnitude below the concentration used for the ssODN repair matrix (2.6 × 10^11^ molecules/μl). We found that pronuclear compared to cytoplasmic microinjection was significantly more efficient to generate KI alleles (Table 1, 12% vs 0% p < 0.01; compare Fig. 2E and F).

### KI efficiency with a dsDNA repair matrix depends on the size of the regions of homology

We obtained 12% KI efficiency with a repair matrix having 500 bp homology regions on each side. We asked whether shorter regions of homology would be sufficient to obtain KI allele with good efficiency. We therefore tested repair matrices with the very same 945 bp non-homology region and either 250 bp or 60 bp homology arms (Fig. 1B). Plasmid concentrations were adjusted according to the number of molecules (1.0 × 10^10^/μl). No KI was obtained with both plasmids although the majority of injected embryos contained indels (Table 1), indicating that, as anticipated from previous work^33^,^34^,^35^, total length of homology in the repair matrix is an important HDR promoting factor in our experimental conditions.

### Cas9 nickase is less efficient than wild-type Cas9 to generate KI alleles

**Figure 3:**
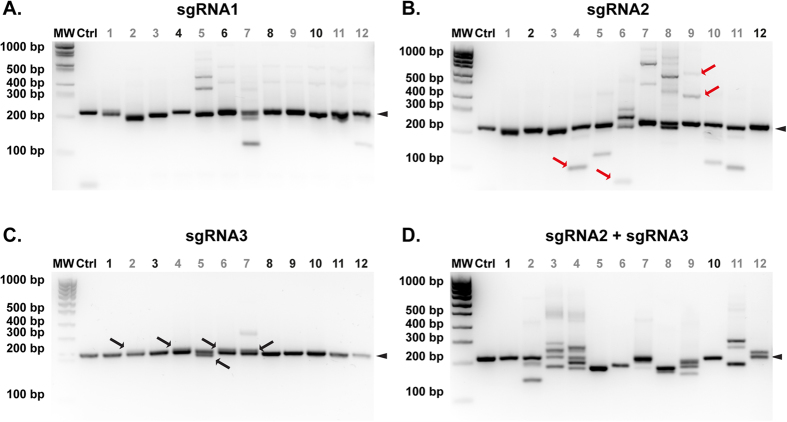
The profile of indels alleles is determined by the sgRNA. Agarose gel electrophoresis analysis of NleF-NleR PCR products of one non-injected embryo (Ctrl) and twelve embryos (lanes 1 to 12) microinjected into the pronucleus with either Cas9 mRNA/sgRNA1 (**A**), Cas9 mRNA/sgRNA2 (**B**), Cas9 mRNA/sgRNA3 (**C**), or Cas9n mRNA/sgRNA2 and 3 (**D**). Large deletions (>100 bp) and insertions (red arrows in B) are frequently observed with Cas9 mRNA/sgRNA2 while small (<10 bp) deletions and insertions (black arrows in C) are frequently observed with Cas9 mRNA/sgRNA3. Arrowheads point to the wild-type band (180 bp). MW: 100 bp molecular weight marker. Embryos with indels and wild type only alleles are labeled in gray and black respectively.

To assess the efficiency of Cas9n and compare it to that of wild type Cas9, we designed two sgRNAs, sgRNA2 and sgRNA3, allowing Cas9n-mediated double nicking centered on the DSB generated by wild type Cas9 and sgRNA1 (Fig. 1A). First of all, we evaluated the efficacy of individual sgRNA to induce DSBs by measuring indel frequency after pronuclear injection with wild-type Cas9 mRNA (Table 1). We found that indel frequency with sgRNA3 (43%) was significantly lower than with sgRNA1 (87%, p < 0.0001) and sgRNA2 (72%, p < 0.01). Strikingly, pronuclear injection with both sgRNA2 and sgRNA3 and Cas9n mRNA generated a high proportion of embryos carrying indels (86%, Table 1), indicating that, despite the moderate efficacy of sgRNA3, similar rates of genome editing at the ATG region of *Nle* were obtained with Cas9/sgRNA1 or Cas9n/sgRNA2 and 3. Interestingly, we noticed that mosaicism was higher with Cas9n compared to wild-type Cas9, as evaluated by the proportion of embryos with more than two alleles (Table 1, 43% vs 0–17% depending on the sgRNA, p < 0.01). Unexpectedly, the type of indels generated seemed to depend on the guide sequence, as sgRNA3 caused only small indels while sgRNA2 caused mostly large indels and combination of the two with Cas9n gave intermediate profiles (Fig. 3). This suggests that the location and/or the nature of the chromosome break may greatly influence the outcome of the repair process and thus the nature of the indels alleles generated by CRISPR/Cas9. Interestingly, our data are consistent with a recent study, performed in human cell lines, showing that the repair outcome of *Streptococcus pyogenes* Cas9-mediated DSBs is not random but determined by the protospacer sequence^36^.

Finally, we compared the KI efficiency with wild-type Cas9 and Cas9n for both ssODN and circular plasmid repair matrices following pronuclear injection (Table 1, compare Fig. 2D with 2 G and 2 F with 2 H). For both ssODN and circular plasmid repair matrices, KI frequency was significantly reduced with Cas9n (14% vs 34%, p < 0.01 and 2% vs 12%; p < 0.05). Our data show that Cas9n is less efficient than wild-type Cas9 to promote HDR events.

### Microinjection conditions do not impact on the fidelity of HDR events but modulate the rate of off-target mutagenesis

Imprecise KI alleles are sometimes generated during DSB repair, leading to unwanted genomic configurations^22^,^37^,^38^. We asked whether increased Cas9 mRNA/sgRNA concentrations or use of Cas9n would affect the fidelity of HDR. We amplified and sequenced *Nle*^*HA*^ and *Nle*^*HA-GFP*^ knock-in alleles generated under various conditions (Supplementary Table S2) and found imprecise KI alleles in about one fourth of the embryos (10 out of 39 for *Nle*^*HA*^ and 2 out of 8 for *Nle*^*HA-GFP*^, Supplementary Fig. S3 and S4). However, no obvious difference in the proportion of imprecise KI alleles was observed when using low versus high Cas9 mRNA/sgRNA concentrations or wild type Cas9 versus Cas9n variant.

We also determined the impact of Cas9 mRNA/sgRNA concentrations and of the site of injection on off-target genome modification. We predicted the off-target sites of sgRNA1 in the mouse genome and selected the three sites (OT1-3) with the highest risk of being edited (Supplementary Fig. S5A). Indels at these loci in injected embryos were first analyzed by size polymorphism of PCR fragments that contain the potential off-target sites (Table 2, Supplementary Fig. S5B). While the three off-target sites similarly differ from the target sequence by three mismatches located outside the seed sequence, indels were observed for OT3 but not OT1 and OT2, suggesting that additional factors regulate the probability of cleavage at off-target sites. Genome editing at the OT3 site was confirmed by sequencing (Supplementary Fig. S5C). Since very small indels (≤5 bp) could be missed by gel electrophoresis analysis, we also sequenced PCR fragments of apparently normal size. No mutations were found for OT1 and OT2, while additional indels at OT3 site were identified in a small proportion of embryos (Table 2, Supplementary Fig. S5C). Similar to on-target mutation, frequency of OT3 editing increased when high Cas9 mRNA/sgRNA concentration was used, independently of the site of injection (Table 2). Importantly, the proportion of off-target mutations amongst embryos with KI alleles was similar after injection with low (2/3 KI out of 11 embryos sequenced for OT3) and high (8/11 KI out of 21 embryos sequenced for OT3) Cas9 mRNA/sgRNA concentration. Collectively, our data indicate that variation in the quantity of injected Cas9 mRNA/sgRNA has a similar effect on the rate of off- and on-target mutations.

## Discussion

Contrasting results in terms of KI efficiency with ssODN^11^,^12^,^37^,^39^,^40^,^41^,^42^ or dsDNA^12^,^17^,^38^,^43^,^44^,^45^ repair matrices have been reported using the Cas9/gRNA system in mouse zygotes. Even for a same locus (5′ region of *Rosa26* intron 1) and a similar type of modification (targeted introduction of a 8–12 kb sequence), sharp differences in KI efficiencies (0% vs 20%) were reported despite experimental conditions leading to a similarly high rate of indels alleles generation (93% vs 74%)^17^,^43^. Thus, efficiency of HDR seems to depend, not only on the locus being targeted and the efficacy of DSBs generation, but also on other factors, the contribution of which remained to be precisely determined. Here, by performing an extensive study in which a single parameter varied while the others were kept constant, we have demonstrated that KI efficiency with a ssODN repair matrix was dependent on the concentration of Cas9mRNA/sgRNA injected but not on the site of injection. Because the rate of indels is also increased under high Cas9mRNA/sgRNA concentration, significant embryo loss may arise when targeting precise mutations into an essential gene, potentially complicating the generation of founder mice and the establishment of the corresponding mouse lines. In a previous report comparing cytoplasmic and pronuclear injections^12^, 20 times more Cas9mRNA/sgRNA and 5–50 times more ssODN repair matrix were used for cytoplasmic injections, making it difficult to discriminate between contributions of the site of injection and the concentration of RNAs and repair matrix. We found that pronuclear injection with high concentration of Cas9mRNA/sgRNA did not affect early embryonic viability or the overall mutation rate compared to cytoplasmic injection. Since HDR with the repair matrix occurs in the nuclear compartment, we propose that pronuclear injection should be favoured when generating KI alleles. Moreover, our data suggest that the site of injection impacts on the genotypic complexity of the founders obtained, since cytoplasmic injection gave a higher proportion of embryos having an indel allele in addition to the desired KI allele. Such situation may complicate the phenotypic characterization of KI alleles when performed directly on F0 mice/embryos.

Consistent with previous reports^12^,^26^, we found a lower KI efficiency with a plasmid compared to ssODN. Obviously, the reduced number of molecules injected and therefore available for HDR could explain, at least in part, this difference. Because injection of large amounts of DNA into mouse zygote is toxic^46^, markedly increasing plasmid concentration cannot be an option. Similarly, using a linear fragment devoid of plasmid sequences may slightly increase the number of dsDNA repair matrix delivered but would likely enhance the incidence of random transgenesis^46^. An interesting alternative is the use of long single-stranded DNA templates since they are less prone to random integration. Currently, length of commercially available ssODN is limited to 200 nt but longer (296–837 nt) ssDNA templates have been produced and successfully used in rodent zygotes to yield KI animals/embryos^23^,^47^. Whether even longer ssDNA templates could be easily produced remains to be determined.

Use of the CRISPR/Cas9 system in zygotes is an efficient and comparatively fast method to generate genetically modified mice. A major issue of the system is potential off-target mutagenesis. Several studies performed in cell lines indicate that Cas9 off-target activity depends on sgRNA sequence^48^,^49^ and that usual programs for predicting off-target mutation sites are not exhaustive^50^,^51^. So far, *in vivo* mutagenesis has been evaluated in a few studies, where off-target mutations were rarely detected following Cas9 mRNA/sgRNA microinjection into mouse zygotes^52^,^53^,^54^. Here, we found mutations at one off-target site with sgRNA1 confirming that off-target mutations should be considered as an important concern when generating CRISPR/Cas9-edited mice. When establishing a CRISPR/Cas9-edited mouse line, one should therefore envisage to performed at least two crosses with wild type mice to segregate the desired on-target modification from genetically unlinked potential off-target mutations. As expected, we found that the rate of off-target mutagenesis increased when high Cas9mRNA/sgRNA concentration was used. Since the proportion of embryos with both on-target KI allele and off-target mutation was unchanged when increasing RNA concentration, using high RNA concentration is expected to improve the recovery of embryos/mice with a KI allele without increasing the risk that these animals carry an off-target mutation.

Alternative approaches based on modified Cas9 nucleases have been developed to increase the specificity of CRISPR/Cas genome editing^27^,^28^,^55^,^56^. In this study, we compared Cas9D10A nickase variant to wild-type Cas9 and showed that it was 2–4 times less efficient to produce KI alleles. Mosaicism and allele complexity in founders represent additional limitations of the CRISPR/Cas9 approach^16^,^26^,^57^,^58^. We found that while the rate of embryos with 3 or more alleles was similar to that previously reported (11–35%) for wild-type Cas9^26^, it appeared globally higher when we injected Cas9n mRNA (23–43%, Table1). Our data therefore suggest that DSBs induction is delayed with Cas9n compared to wild-type Cas9, probably because two nuclease/sgRNA complexes instead of one need to be recruited locally on the chromosome to induce a break and because individual nicks can be efficiently repaired by other pathways such as the base excision repair pathway. Our data indicate that, when considering using Cas9n to generate genetically modified mice with lower risk of unwanted off-target mutations, its decreased efficiency for KI allele production and its tendency to generate a higher proportion of mosaic founders should be taken into account.

Finally, we found unexpected differences in the profile of indel alleles generated by three sgRNAs. The reasons for such differences in the repair of very close DSBs (less than 34 bp) are currently unknown. In any case, this observation stresses the fact that our understanding of how DSBs are being repaired in the mouse embryo is incomplete.

In conclusion, CRISPR/Cas9 has revolutionized the field of genetically modified mice generation and the genome editing toolbox is expanding very fast, offering new options to produce edited mice in a more versatile^59^, safe^60^,^61^,^62^ and efficient manner^63^,^64^. In the future, the benefit of these new developments on KI alleles production will need to be systematically and carefully evaluated.

## Materials and Methods

### Animals

(C57BL/6xSJL/J) F1 female mice for oocyte generation were purchased from Janvier, France. They were mated with CD1-IGS male mice purchased from Charles River Laboratories, France. All experiments were conducted at the Institut Pasteur according to the French and European regulations on care and protection of laboratory animals (EC Directive 86/609, French Law 2001-486 issued on June 6, 2001) and were approved by the Institut Pasteur ethics committee (n° 2016-016).

### Preparation of Cas9 mRNA and sgRNA

Cas9 mRNA and sgRNAs were prepared according to the online protocol from the Feng Zhang lab (http://www.genome-engineering.org/crispr/). For each guide sequence, a pair of oligonucleotides were annealed and cloned into BbsI-digested px330 expression vector. sgRNAs, wild-type Cas9 and Cas9n DNA templates containing a T7 promoter were obtained by PCR amplification on px330 and px335 plasmids using high fidelity TaKaRa LA Taq. After sequencing, PCR products were used as templates for *in vitro* transcription using MEGAshortscript T7 Transcription and mMESSAGE mMACHINE T7 Ultra Kits (Life Technologies, Carlsbad, CA, USA). Cas9 mRNA and sgRNAs were then purified using LiCl/ethanol precipitation and resuspended in Brinster’s Buffer (10 mM Tris-HCl pH 7.5; 0.25 mM EDTA). Oligonucleotides used for cloning and amplification are listed in Supplementary Table S3.

### Repair matrices

ssODN repair matrices were ordered as PAGE-purified Ultramer DNA oligonucleotides from Integrated DNA Technologies. The NleGFP500 plasmid was obtained from GeneScript as a 1,963 bp gene synthesis fragment inserted into the pUC57 expression vector. NleGFP250 and NleGFP60 plasmids were derived from the NleGFP500 plasmid by PCR amplification (Table S3) and cloning into PCR II-topo vector using a TOPO TA cloning Kit (Invitrogen). All plasmids were prepared using the QIAfilter Plasmid Midi Kit (Qiagen) and verified by sequencing before injection.

### Microinjection into mouse zygotes

Fertilized eggs were obtained from superovulated 3 week-old (C57BL/6xSJL/J) F1 females submitted to intra-peritoneal injection of pregnant mare serum gonadotropin (Chronogest PMSG 2.5 units/mouse, Centravet) followed by human chorionic gonadotropin (Chorulon hCG, 5 units/mouse, Centravet) 42–48 hours later and then mated overnight with CD1 males. One-cell-stage embryos were harvested 20–22 hours after human chorionic gonadotropin injection. The ampullary region of oviducts was placed at 37 °C in EmbryoMax M2 medium with Phenol Red (Millipore Bioscience) containing bovine hyaluronidase Type IV (Sigma) at 0.5 mg/ml and punctured with forceps to release the clutch of eggs. After 1 minute, the cumulus cells were dissociated and the eggs washed by transfer through successive passages (four to six) of fresh EmbryoMax M2 Medium with Phenol Red. Fertilized eggs displaying two pronuclei and polar bodies were cultured in a Nunclon surface 4 well plate (NUNC) in 400 μl of EmbryoMax KSOM + AA with D-Glucose with Phenol Red (Millipore Bioscience) in 8% CO2 at 37 °C. For injection, zygotes were transferred to a depression slide in EmbryoMax M2 medium overlaid with embryo-tested mineral oil (Sigma) and microinjected using an Olympus IMT-2 inverted microscope equipped with a Leitz micromanipulator. RNAs and DNAs were mixed and diluted into Brinster’s Buffer (10 mM Tris-HCl pH 7.5; 0.25 mM EDTA). Microinjection mixtures were dropped on the depression slide and aspirated with the injection pipette for pronuclear or cytosolic injection. Embryos were then cultured in EmbryoMax KSOM + AA with D-Glucose with Phenol Red at 8% CO2 at 37 °C for 72 h. All injections were performed by a single person (S.V.-P.).

### On-target PCR amplification

Embryos were transferred into 5 μl embryo lysis buffer (10 mM TrisHCl pH 8; 50 mM KCl; 0.01% gelatin) supplemented with 300 μg/mL proteinase K (Eurobio) and incubated at 56 °C for 60 min. Proteinase K was then inactivated at 95 °C for 15 min. For the *Notchless* locus, whole lysates were PCR-amplified using 3.125 units of Taq DNA polymerase (MP biomedicals) and 0.315 units of high fidelity LA Taq (TaKaRa) under the following conditions: 95 °C for 30 s, 40 cycles at 98 °C for 30 s, 68 °C for 30 s, 72 °C for 2 min 50 s, 72 °C for 10 min and using ExtF and ExtR primers (Supplementary Table S3) located 930 bp 5′ and 1313 bp 3′ to *Notchless* ATG respectively. Nested PCR was then performed on 1/25 of the first PCR product using either HAF and HAR or NleF and NleR primers under the following conditions: 95 °C for 30 s, 25 cycles at 95 °C for 30 s, 58 °C for 30 s, 72 °C for 60 s, 72 °C for 10 min. For the *Sox2* locus, whole lysates were PCR-amplified using 1.0 unit of Taq DNA polymerase (MP biomedicals) under the following conditions: 95 °C for 30 s, 25 cycles at 95 °C for 30 s, 58 °C for 30 s, 72 °C for 60 s, 72 °C for 10 min and using Sox2ExtF and Sox2ExtR primers (Supplementary Table S3). Nested PCR was then performed on 1/25 of the first PCR product using either Sox2intF and HAR or Sox2intF and Sox2intR primers under the following conditions: 95 °C for 30 s, 25 cycles at 95 °C for 30 s, 58 °C for 30 s, 72 °C for 60 s, 72 °C for 10 min.

### Analysis of Off-Target sites

Off-target sites of sgRNA1 were predicted and scored using algorithms from the Feng Zhang lab (http://crispr.mit.edu/). These algorithms provide a list of potential off-target sites with up to four mismatches to the guide and a canonical (NGG) or non-canonical (NAG) PAM sequence. The risk score is calculated based on mismatch position and PAM canonicity. No off-target site with less than three mismatches was found for sgRNA1. The three sites with the highest risk score (OT1, OT2 and OT3) display a canonical PAM sequence and three mismatches located outside the 10 nt seed region of the guide (proximal to the PAM). OT1 and OT2 are two copies of a repeated sequence within the mouse *Firre* locus differing only by a few SNPs. We designed primers allowing to amplify both sites at the same time and verified by sequencing that both sites were indeed amplified. PCR was performed on 1/4 of the lysate using 1.0 unit of Taq DNA polymerase (MP biomedicals) under the following conditions: 95 °C for 30 s, 25 cycles at 95 °C for 30 s, 58 °C for 30 s, 72 °C for 60 s, 72 °C for 10 min and using OT1ExtF and OT1ExtR primers for OT1 and OT2 sites or with OT3ExtF and OT3ExtR for OT3 (Supplementary Table S3). Nested PCR was then performed on 1/25 of the first PCR product using OT1intF and OT1intR or OT3intF and OT3intR primers under the following conditions: 95 °C for 30 s, 25 cycles at 95 °C for 30 s, 58 °C for 30 s, 72 °C for 60 s, 72 °C for 10 min.

### Mutation detection by agarose gel electrophoresis

PCR products were separated by electrophoresis on either 4% high-resolution (NuSieve 3:1 agarose) or 2% high-resolution (MetaPhor agarose) agarose gels allowing the detection of ≥ 5 bp size polymorphisms. Embryos harbouring a knock-in allele were identified according to the presence of bands of the expected sizes for both general (NleF-NleR or Sox2intF-Sox2intR) and allele-specific (HAF-HAR or GFPF-GFPR or Sox2intR-HAR) amplicons (Fig. 1C). On-Target NHEJ alleles were identified based on a size for NleF-NleR or Sox2intF-Sox2intR amplicons differing from that of wild-type (180 bp or 270 bp) and KI (210 bp for HA-Nle, 1.1 kbp for GFP-Nle, 300 bp for HA-Sox2) alleles. Off-Target NHEJ alleles were identified based on a size for OT1intF-OT1intR or OT3intF-OT3intR amplicons differing from that of the wild-type (230 bp) alleles. Embryos yielding only one PCR product were considered homozygous and those displaying 3 or more alleles were considered mosaic. Because very small indels and indels encompassing a region corresponding to one of the primer used for genotyping may be missed by our analysis, our evaluation of indel and mosaicism rates may be systematically underestimated.

### Mutation detection by sequencing

Sequencing of PCR products was performed using the Applied Biosystems 3130 Analyzer and the BigDye^®^ Terminator v3.1 Cycle Sequencing Kit (ThermoFisher) according to manufacturer’s instructions. Wild type *Notchless* and OT3 loci were sequenced on PCR products obtained on DNAs from one (C57BL/6xSJL/J) F1 female and six CD1-IGS males. Sequencing of knock-in or NHEJ alleles was performed on nested PCR products generated from embryo lysates. For some embryos, the OT3 PCR product was sub-cloned using a TOPO TA Cloning Kit (ThermoFisher) and DH5α competent cells (ThermoFisher) according to manufacturer’s instructions. Cloned fragments were PCR-amplified using BSBI and BSBII primers (Supplementary Table S3) under the following conditions: 95 °C for 30 s, 25 cycles at 95 °C for 30 s, 60 °C for 30 s, 72 °C for 60 s, 72 °C for 10 min before sequencing.

### Statistical analysis

All p-values were calculated using Fisher’s exact test.

## Additional Information

**How to cite this article**: Raveux, A. *et al*. Optimization of the production of knock-in alleles by CRISPR/Cas9 microinjection into the mouse zygote. *Sci. Rep.* **7**, 42661; doi: 10.1038/srep42661 (2017).

**Publisher's note:** Springer Nature remains neutral with regard to jurisdictional claims in published maps and institutional affiliations.

## Supplementary Material

Supplementary Figures

## Figures and Tables

**Table 1:** Summary of CRISPR/Cas9-mediated *Nle* exon 1 mutations obtained after mouse zygotes microinjection.

sgRNA/Cas9 variant	HDR matrix (homol. arm)	site of injection	Cas9/sgRNA (ng/μl)	HDR matrix (ng/μl)	Nb exp.	Nb embryos*/Nb injected (%)	Nb analyzed	KI (%)	Indels** (%)	WT*** (%)	>2 alleles (%)
sgRNA1/wild-type	ssODN	Pronucleus	5/2.5	2	1	59/80 (74)	59	3 (5)	7 (12)	49 (83)	2 (3)
				20	2	81/142 (57)	81	12 (15)	12 (15)	59 (73)	2 (2)
				40	1	45/65 (69)	37	1 (3)	9 (24)	27 (73)	0 (0)
			100/50	20	2	57/114 (50)	50	17^a^ (34)	27 (54)	9 (18)	8 (16)
		Cytosol	5/2.5	20	2	74/115 (64)	74	10 (14)	33 (45)	35 (47)	10 (14)
			100/50	20	2	58/106 (55)	53	21^b^ (40)	34 (64)	10 (19)	15 (28)
	plasmid (500 bp)	Pronucleus	100/50	4	2	51/97 (53)	42	0 (0)	32 (76)	10 (24)	2 (5)
				40	3	59/173 (34)	52	6 (12)	32 (62)	17 (33)	8 (15)
				80	2	49/134 (29)	45	1^c^ (2)	28 (62)	16 (35)	2 (4)
		Cytosol	100/50	40	2	96/175 (55)	81	0 (0)	56 (69)	25 (31)	8 (10)
	plasmid (250 bp)	Pronucleus	100/50	46^††^	3	73/192 (38)	65	0 (0)	50 (77)	15 (23)	8 (12)
	plasmid (60 bp)	Pronucleus	100/50	43^††^	2	53/178 (30)	47	0 (0)	36 (77)	11 (23)	5 (11)
	none	Pronucleus	100/50	0	2	47/74 (64)	46	NA	40 (87)	6 (13)	8 (17)
sgRNA2/wild-type	none	Pronucleus	100/50	0	1	35/60 (58)	32	NA	23 (72)	9 (28)	4 (13)
sgRNA3/wild-type	none	Pronucleus	100/50	0	1	38/59 (64)	38	NA	16 (42)	22 (58)	0 (0)
sgRNA2 + sgRNA3/nickase	none	Pronucleus	100/50^†^	0	2	58/103 (56)	56	NA	48 (86)	8 (14)	24 (43)
	ssODN	Pronucleus	100/50^†^	20	2	69/109 (63)	63	9^a^ (14)	52 (82)	9 (14)	24 (38)
	plasmid (500 bp)	Pronucleus	100/50^†^	40	4	101/275 (37)	87	2 (2)	51 (59)	36 (41)	20 (23)

Cas9 mRNA, sgRNA and repair matrix were injected into the cytoplasm or the male pronucleus at the indicated concentrations. Embryos surviving the injection were cultured and embryos that developed up to 8-cell stage and beyond were analysed by PCR and migration of the amplification products on agarose gels.

*8-cell stage to early blastocyst; **: because indels ≤5 bp could be missed by gel electrophoresis analysis, the number of indels is likely underestimated; ***WT corresponds to embryos for which no edited alleles was detected.

^†^50 ng/μl of each sgRNA, ^††^plasmid concentration was adjusted to an equivalent of 1.0 × 10^10^ molecules/μl.

^a^Including two homozygous KI embryos. ^b^Including four homozygous KI embryos. ^c^Including one homozygous KI embryo.

**Table 2:** Summary of CRISPR/Cas9-mediated off-target mutations obtained after mouse zygotes microinjection.

Site of injection	Cas9/sgRNA (ng/μl)	ssODN (20 ng/μl)	Nb exp.	Nb embryos*/Nb injected (%)	Indels alleles at off-target loci identified by						
					size polymorphism				sequencing^a^		
					Nb analyzed	OT1 (%)	OT2 (%)	OT3 (%)	OT1	OT2	OT3
Pronucleus	5/2.5	Yes	1	40/95 (42)	39	0 (0)	0 (0)	1^b,c^ (3)	0/10	0/10	1/10 ^c^
	100/50	Yes	1	22/56 (39)	21	0 (0)	0 (0)	9^b,c^ (43)	0/10	0/10	2/12 ^c^
	100/50	No	2	46/78 (59)	37	0 (0)	0 (0)	15 (41)	ND		
Cytosol	5/2.5	No	2	55/85 (65)	53	0 (0)	0 (0)	4 (8)	ND		
	100/50	No	2	65/98 (66)	63	0 (0)	0 (0)	31 (48)	ND		

Cas9 mRNA and sgRNA1 were injected into the cytoplasm or the male pronucleus with or without ssODN repair matrix at 20 ng/μl. Embryos surviving the injection were cultured and embryos that developed up to 8-cell stage and beyond were analysed by PCR and migration of the amplification products on agarose gels.

*8-cell stage to early blastocyst.

^a^Sequencing of normal size PCR fragments was performed for a subset of injected embryos.

^b^OT3 indel alleles were confirmed by sequencing.

^c^Embryos were further analysed for the presence of a *Nle*^*HA*^ KI allele.

ND: not done.
