# Supplementary material for: Optimization of the production of knock-in alleles by CRISPR/Cas9 microinjection into the mouse zygote
Source: Sci Rep. 2017 Feb 17;7:42661. doi: 10.1038/srep42661 (PMC5314402; doi:10.1038/srep42661)
Supplement: Supplementary Figures [file srep42661-s1.pdf]

Supplementary Information

Supplementary Figures S1 to S5 and Tables S1 to S3.

Optimization of the production of knock-in alleles by CRISPR/Cas9 microinjection into the mouse zygote

Aurélien Raveux, Sandrine Vandormael-Pournin and Michel Cohen-Tannoudji

Institut Pasteur, CNRS, Unité de Génétique Fonctionnelle de la Souris, UMR 3738, Department of Developmental & Stem Cell Biology, 25 rue du docteur Roux, F-75015 Paris Cedex.

Corresponding author: Michel Cohen-Tannoudji, Unité de Génétique Fonctionnelle de la Souris, Department of Developmental & Stem Cell Biology, 25 rue du docteur Roux, F-75015 Paris, France; E-mail: m-cohen@pasteur.fr; Phone: 33 1 45 68 84 86; Fax: 33 1 45 68 86 34, web site: https://research.pasteur.fr/en/team/group-michel-cohen-tannoudji/.

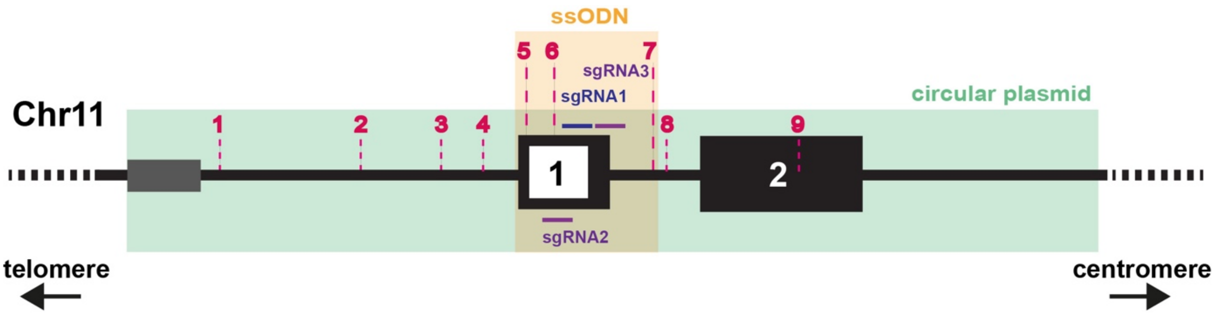

SNP	1	2	3	4	5	6	7	8	9
Position	82908717	82908564	82908489	82908431	82908406	82908375	82908291	82908274	82908146
NCBI m37	T	T	G	C	T	C	A	G	A
C57BL6xSJL/J	T/C	T/C	G/A	C/T	T/C	C/T	A/C	G/A	A/C
CD1 #1	T	T	G	C	T	C	A	G	A
CD1 #2	T/C	T/C	G/A	C/T	T/C	C/T	A/C	G/A	A/C
CD1 #3	T/C	T/C	G/A	C/T	T/C	C/T	A/C	G/A	A/C
CD1 #4	T/C	T/C	G/A	C/T	T/C	C/T	A/C	G/A	A/C
CD1 #5	T	T	G	C	T	C	A	G	A
CD1 #6	T/C	T/C	G/A	C/T	T/C	C/T	A/C	G/A	A/C

Figure S1 : Single nucleotides polymorphisms (SNPs) around *Nle* exon 1.
Tail DNAs from one (C57BL/6xSJL/J) F1 female and six CD1-IGS males were amplified using primers WTF1 and GFPR (169bp 5' and 522bp 3' to the ATG respectively) and WTF2 and GFP250R (500bp 5' and 250bp 3' to the ATG respectively), and sequenced using GFP60F and GFP60R primers respectively (see Supplementary Table S1 for primer sequences). 9 SNPs compared to the C57BL6 reference genome (NCB1 m37) were identified and their positions respective to exon 1 and 2 and to the regions covered by the ssODN matrix (orange box) and the plasmid repair matrix (green box) are indicated on the scheme. The grey box located at the telomeric end of the green box represent a small GC and CA repeat region that could not be sequenced properly.
8 out of the 9 SNPs were already present in dbSNP Build 142. SNP n°1 has been identified in this study. (C57BL/6xSJL/J)F1 females and 4 out of the 6 CD1-IGS males are heterozygous at all SNP positions.

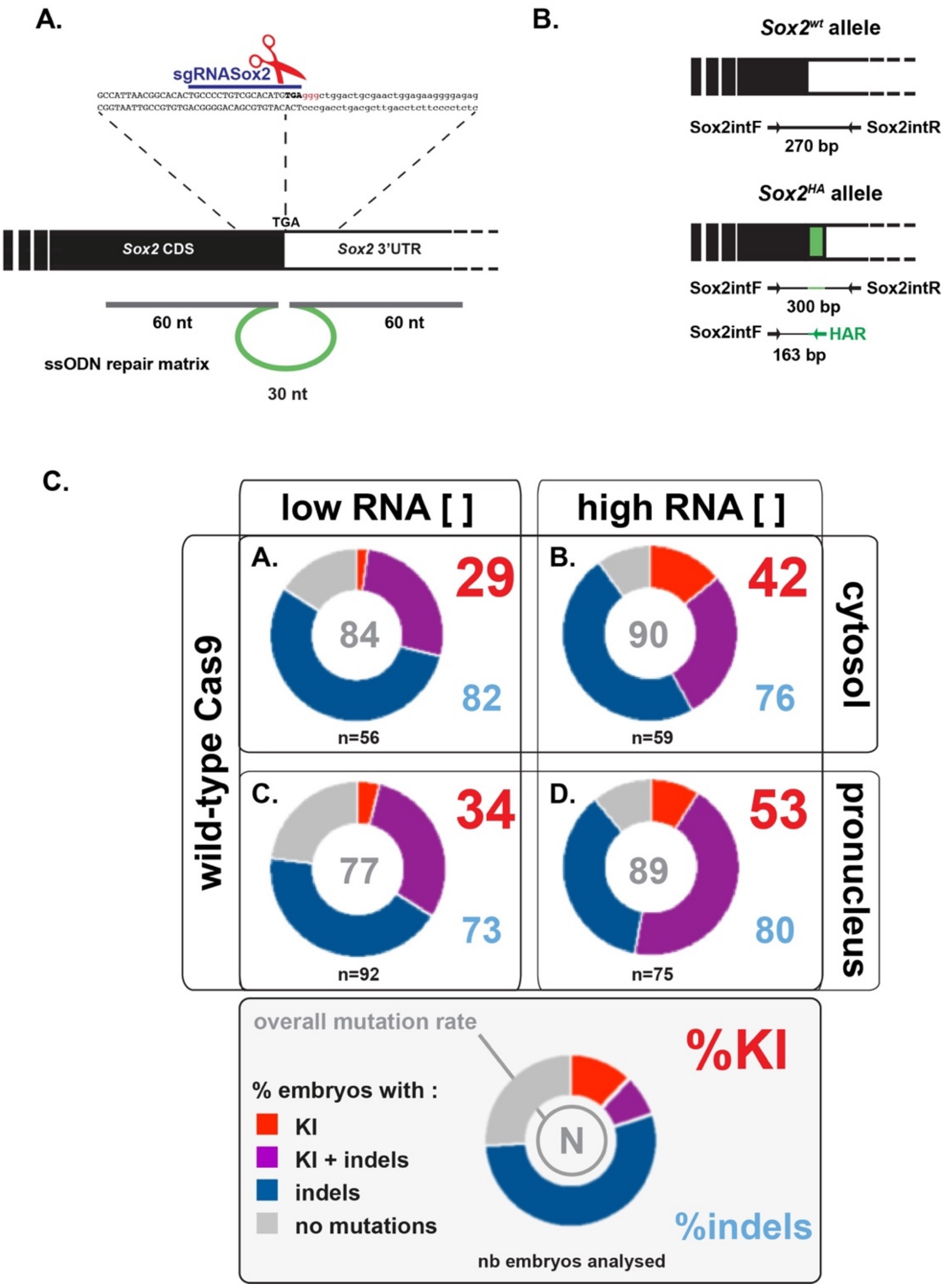

Figure S2 : Comparison of the effect of Cas9/sgRNA consentration and the site of injection on KI efficiency at the *Sox2* locus.

A. Schematic representation of the end of *Sox2* coding region, sgRNA sequence and ssODN repair matrix. *Sox2* TGA stop codon is indicated in bold, the sgRNA-targeting sequence is underlined, and the protospacer adjacent motif sequences is labelled in red. Cas9 nuclease cuts DNA at position -3 bp from the protospacer adjacent motif. The ssODN repair matrix contains 60 nucleotides of homology flanking both sides of the DSB generated by sgRNASox2. B. Schematic representation of *Sox2^{wt}* and *Sox2^{HA}* alleles. Positions of the primers and sizes of the PCR fragments are indicated below each allele. C. Each circle represents the proportion of embryos with KI, indels, KI and indels, or wild-type only alleles for a given condition. The overall mutation rate, which corresponds to the proportion of embryos displaying at least one edited allele, is indicated at the center of each disk. Because some embryos contained both KI and indels alleles, the overall mutation rate can be less than the sum of the rates of KI and indels alleles. High concentration ([ ]) correspond to 100 ng/µl of Cas9 mRNA and 50 ng/µl of sgRNA while low [ ] corresponds to 5 ng/µl of Cas9 mRNA and 2.5 ng/µl of sgRNA. ssODN were injected at 20 ng/µl.

A.

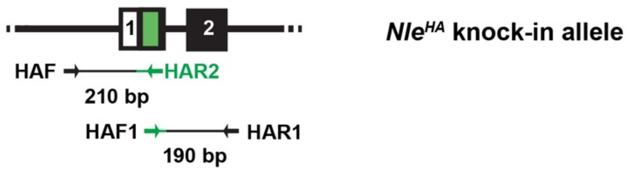

B.

pronuclear, low RNA concentration, Cas9

```
Low #02    CCTCGGTTCTCCTCCACGTGGGGACGCAGGATGGGGTACCCATATGATGTTCCAGATTATGCTGCGGCGGCGGTGGTGGTAGGTGTACGCGCACGGGGC
Low #04    CCTCGGTTCTCCTCCACGTGGGGACGCAGGATGGGGTACCCATATGATGTTCCAGATTATGCTGCGGCGGCGGTGGTGGTAGGTGTACGCGCACGGGGC
Low #05    CCTCGGTTCTCCTCCACGTGGGGACGCAGGATGGGGTACCCATATGATGTTCCAGATTATGCTGCGGCGGCGGTGGTGGTAGGTGTACGCGCACGGGGC
Low #08    CCTCGGTTCTCCTCCACGTGGGGACGCAGGATGGGGTACCCATATGATGTTCCAGATTATGCTGCGGCGGCGGTGGTGGTAGGTGTACGCGCACGGGGC
Low #09    CCTCGGTTCTCCTCCACGTGGGGACGCAGGATGGGGTACCCATATGATGTTCCAGATTATGCTGCGGCGGCGGTGGTGGTAGGTGTACGCGCACGGGGC
Low #10    CCTCGGTTCTCCTCCACGTGGGGACGCAGGATGGGGTACCCATATGATGTTCCAGATTATGCTGCGGCGGCGGTGGTGGTAGGTGTACGCGCACGGGGC

Low #01    CCTCGGTTCTCTTCCACGTGGGGACGCAGG----------------ATGTTCCAGATTATGCTGCGGCGGCGGTGGTGGTAGGTGTACGCGCACGGGGC
Low #03    CCTCGGTTCTCCTCCACGTGGGGACGCA------GGTACCCATATGATGTTCCAGATTATGCTGCG [NA]
Low #06    CCCCGGTTCTCCTCCACGTGGGGACGCAGGATGGGGT--CCATATGATGTTCCAGATTATGCTGCG [NA]
Low #07    CCTCGGTTCTCCTCCACGTGGGGACGCAGGATATCTGATATAAATATGATGTTCCAGATTATGCTGCGGCGGCGGTGGTGGTAGGTGTACGCGCACGGGGC
```

pronuclear, High RNA concentration, Cas9

```
High#01    CCTCGGTTCTCCTCCACGTGGGGACGCAGGATGGGGTACCCATATGATGTTCCAGATTATGCTGCGGCGGCGGTGGTGGTAGGTGTACGCGCACGGGGC
High#04    CCTCGGTTCTCCTCCACGTGGGGACGCAGGATGGGGTACCCATATGATGTTCCAGATTATGCTGCGGCGGCGGTGGTGGTAGGTGTACGCGCACGGGGC
High#05    CCTCGGTTCTCCTCCACGTGGGGACGCAGGATGGGGTACCCATATGATGTTCCAGATTATGCTGCGGCGGCGGTGGTGGTAGGTGTACGCGCACGGGGC
High#06    CCTCGGTTCTCCTCCACGTGGGGACGCAGGATGGGGTACCCATATGATGTTCCAGATTATGCTGCGGCGGCGGTGGTGGTAGGTGTACGCGCACGGGGC
High#07    CCTCGGTTCTCCTCCACGTGGGGACGCAGGATGGGGTACCCATATGATGTTCCAGATTATGCTGCGGCGGCGGTGGTGGTAGGTGTACGCGCACGGGGC
High#08    CCTCGGTTCTCCTCCACGTGGGGACGCAGGATGGGGTACCCATATGATGTTCCAGATTATGCTGCGGCGGCGGTGGTGGTAGGTGTACGCGCACGGGGC
High#09    CCTCGGTTCTCCTCCACGTGGGGACGCAGGATGGGGTACCCATATGATGTTCCAGATTATGCTGCGGCGGCGGTGGTGGTAGGTGTACGCGCACGGGGC
High#10    CCTCGGTTCTCCTCCACGTGGGGACGCAGGATGGGGTACCCATATGATGTTCCAGATTATGCTGCGGCGGCGGTGGTGGTAGGTGTACGCGCACGGGGC

High#02-1  CCTCGGTTCTCCTCCACGTGGGGAC--AGGATGGGGTACCCATATGATGTTCCAGATTATGCTGCGGCGGCGGTGGTGGTAGGTGTACGCGCACGGGGC
High#02-2  C-TCGG-TCTCCTTCACGTGGGGAC---------GG--CCCATTCGATGTTCCAGATTATGCTGCG [NA]

High#03-1  CCTCGGTTCTCCTCCACGTGGGGACGCAGGATGGGGTACCCATATGATGTTCCAGATTATGCTGCGGCGGCGGTGGTGGTAGGTGTACGCGCACGGGGC
High#03-2  CCTCGG--------------------CAGGATGGGGTACCCATATGATGTTCCAGATTATGCTGCGGCGGCGGTGGTGGTAGGTGTACGCGCACGGGGC
```

pronuclear, High RNA concentration, Cas9D10A

```
Nick#02    CCTCGGTTCTCCTCCACGTGGGGACGCAGGATGGGGTACCCATATGATGTTCCAGATTATGCTGCGGCGGCGGTGGTGGTAGGTGTACGCGCACGGGGC
Nick#03    CCTCGGTTCTCCTCCACGTGGGGACGCAGGATGGGGTACCCATATGATGTTCCAGATTATGCTGCGGCGGCGGTGGTGGTAGGTGTACGCGCACGGGGC
Nick#06    CCTCGGTTCTCCTCCACGTGGGGACGCAGGATGGGGTACCCATATGATGTTCCAGATTATGCTGCGGCGGCGGTGGTGGTAGGTGTACGCGCACGGGGC
Nick#07    CCTCGGTTCTCCTCCACGTGGGGACGCAGGATGGGGTACCCATATGATGTTCCAGATTATGCTGCGGCGGCGGTGGTGGTAGGTGTACGCGCACGGGGC
Nick#08    CCTCGGTTCTCCTCCACGTGGGGACGCAGGATGGGGTACCCATATGATGTTCCAGATTATGCTGCGGCGGCGGTGGTGGTAGGTGTACGCGCACGGGGC

Nick#01-1  CCTCGGTTCTCCTCCACGTGGGGACGCAGGTTGGGGTACCCATATGATGTTCCAGATTATTCTGCGGCGGCGGTGGTGGTAGGTGTACGCGCACGGGGC
Nick#01-2  CCTC----------CACGTGGGGACGCAGGATGGGGTACCCATATGATGTTCCAGATTATTCTGCGGCGGCGGTGGTGGTAGGTGTACGCGCACGGGGC

Nick#04-1  CCTCGGTTCTCCTCCACGTGGGGACGCAGGATGGGGTACCCATATGATGTTCCAGATTATGCTGCGGCGGCGGTGGTGGTAGGTGTACGCGCACGGGGC
Nick#04-2  CCTCGGTTCTCCTCCACGTGGGGACGCAGGATGGGGTACCCATATGATGTTCCAGATTATGCTGCGGCGGCG-------------------CACGGGGC

Nick#05-1  CCTCGGTTCTCCTCCACGTGGGGACGCAGGATGGGGTACCCATATGATGTTCCAGATTATGCTGCGGCGGCGGTGGTGGTAGGTGTACGCGCACGGGGC
Nick#05-2  CCTCGGTTCTCTTCCACGTGGGGACGCAGG-----GTACCCATATGATGTTCCAGATTATGCTGCGGCG
                                 TGGGGACGCAGG-----GTACCCATATGATGTTCCAGATTATGCTGCG [NA]

Nick#09   CCTCGG TTCTCCTCCACGTGGGGACGCAGGATGGGGTACCCATATGATGTTCCAGATTATGCTGCGGCGGCGGTGGTGGTAGGTGTACGCGCACGGGGC
             AAAGTACACATGGTATTACAGTTTGTTTGCAAG...........CCCATGTAAATGCTCACCAAAAGGTGACTTCAGCTGAGGAGGAG
```

insertion of 250 bp MuERV-L pol (5’ and 3’ truncated)

C.

```
Nick#09
CCTCGGAAAGTACACATGGTATTACAGTTTGTTTGCAAGAAGAAATGGCCAGATGTACGATTATACACTGACTCATGGGCTGTAGCCAATGGATTGGCTGGATGGT
CAGGGACTTGGAAAGATCACAATTGGAAAATTGGTGAGAAAGACATCTGGGGAAGAAGTATGTGGATAGATCTCTCCAAATGGGCAAAGGATGTGAAGATATTTGT
GTCCCATGTAAATGCTCACCAAAAGGTGACTTCAGCTGAGGAGGAGTTCCACGTGGGGACGCAGGATGGGGTACCCATATGATGTTCCAGATTATGCTGCGGCGGC
GGTGGTGGTAGGTGTACGCGCACGGGGC
```

Figure S3 : Sequencing of *Nle^HA* KI alleles.

A. Schematic representation of the *Nle^HA* KI allele indicating the position of the primers used to generate the PCR products that have been sequenced and the size of the amplicons. B. Sequences of the *Nle^HA* KI alleles from embryos injected under different conditions. The HA-tag sequence is underlined. Insertions, deletions and substitutions are labeled in red or green. Most of the imprecise KI events consisted in small indels in the HA tag region. For some embryos, no HAF1-NleR PCR product were recovered probably because of the deletion in the sequence complementary to the HAF1 primer. As a consequence, sequence 3’ to the HA tag was not analyzed (NA) for these embryos. We found one duplication of the HA tag (Nick#05) and a transposon insertion (Nick#09). C. Complete sequence of the insertion identified in the embryo nick#09.

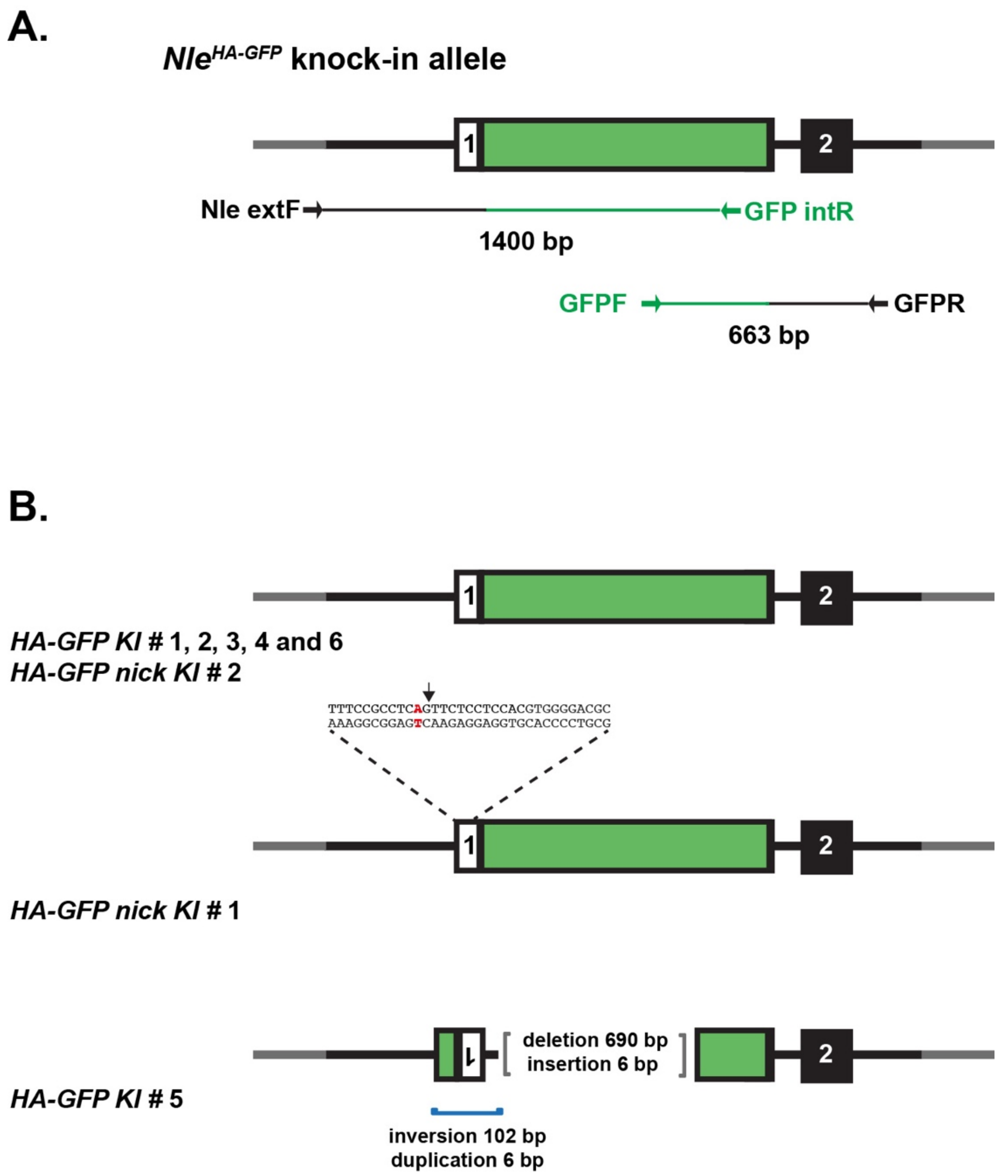

C.

HA-GFP KI#5

TGCCCGGCGCGACATGGATTTA*ACGTCATAGGGATAGCCAGCGTAATCTGGAACATCGTATGGGTACCCCATCCTGCG*
*TCCCCACGTGGAGGAGAACCGAGGCGGAAAGACGGAGCGTTAAATC*TCGTGCCAACGAGAAGCGCGATCACATGGTCC
TGCTGGAGTTCGTGACCGCCGCCGGGATCACTCTCGGCATGGACGAGCTGTACAAGGGAGTGGACCCCCTGGACGAG
ATGGCATCCCTGGACAGCCTGGACTTCCTGGTCCTCCTGGTCCCCCTGGGCCCCCCGGACCCCCTGGCCTTGGAGGAG
CCGGAGGAAGCGGAGGAGGAAGCGGAGCCGCGGCGGCGGTGGTGGTAGGTGTACGCGCACGGGGCGGGGATGCCGGGT
CTCCGCGTG

Figure S4 : Sequencing of *Nle^HA-GFP^* KI alleles.

A. Schematic representation of the *Nle*$^{HA\text{-}GFP}$ KI allele indicating the position of the primers used to generate the PCR products that have been sequenced and the size of the amplicons. B. Sequence of the *Nle*$^{HA\text{-}GFP}$ KI alleles from embryos injected in the pronucleus with high RNA concentration of Cas9 mRNA/sgRNA (HA-GFP KI#1-6) or Cas9n mRNA/sgRNA (HA-GFP nick KI#1-2). HA-GFP nick KI #1 has a point mutation at the vicinity of sgRNA2 nicking site (arrow). HA-GFP KI #5 presents several rearrangements in the HDR region. C. Sequence of the rearranged HA-GFP KI #5 allele. The inverted region is indicated in italics. The 6 bp insertion is labeled in red.

A.

	Sequence	PAM	Risk	Locus
	GTGGGGACGCAGGATGGCGG	CGG		
OT1	.A..A..A............	AGG	2.54	ChrX:47925998
OT2	.A..A..A............	AGG	2.54	ChrX:47961461
OT3	T....A.T............	AGG	1.57	Chr2:118424170
	....T...A..........C	CGG	0.82	Chr5:121125482
	A..A.............G..	AGG	0.68	Chr13:100127075
	....T...T.........T.	GGG	0.61	Chr18:57107171
	....A..T.........G..	CGG	0.60	Chr2:33533762
	....T..A.........T..	AGG	0.60	Chr8:35658266
	...T.C...........A..	AGG	0.37	Chr14:40077574
	.G........C......G..	GGG	0.36	Chr4:118778978
	.......GA........G..	TGG	0.34	Chr2:31267876
	........TG.......G..	TGG	0.30	Chr14:13392252
	C.........C..G......	CGG	0.25	Chr11:115243405
	..........T.......CC	AGG	0.20	Chr2:103985087
	.........G.G.C......	AGG	0.18	Chr10:7676155
	..C...........T....T.	CGG	0.16	Chr7:19928075
	.....C.......C....C.	TGG	0.09	Chr16:95038801
	.......A.....G...A..	GGG	0.08	Chr12:107155672
	........C....C...T..	TGG	0.05	Chr14:79063603
	.............GA...A.	GGG	0.03	Chr9:61882428

B.

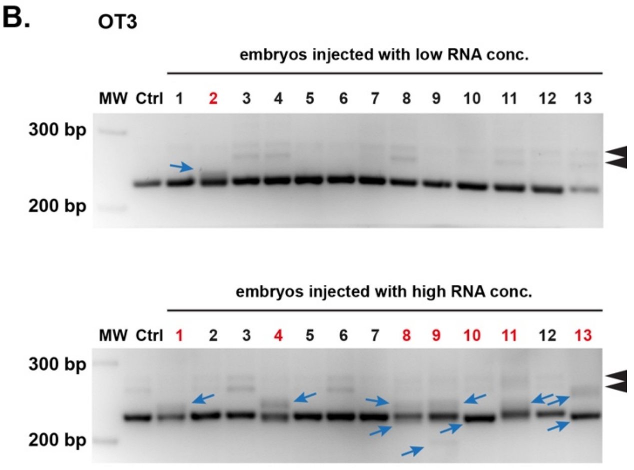

C.

```
Low #2
WT1-CTGCGGAAGGGCGAGAGCAAGTATTGGGAATGCAGGATGGCGGAGGCGAGGTAAGGACCGGACGCAAATT
WT2-CTGCGGAAGGGCGAGAGCAAGTATTGGGAATGCAGGATGGCGGAGGCGAGGTAAGGACCGGACGCAAGTT
Dl1-CTGCGGAAGGGCGAGAGCAAGTATTGGGAATGCAGGATG-CGGAGGCGAGGTAAGGACCGGACGCAAATT

High #1
WT1-CTGCGGAAGGGCGAGAGCAAGTATTGGGAATGCAGGATGGCGGAGGCGAGGTAAGGACCGGACGCAAATT
WT2-CTGCGGAAGGGCGAGAGCAAGTATTGGGAATGCAGGATGGCGGAGGCGAGGTAAGGACCGGACGCAAGTT
In1-CTGCGGAAGGGCGAGAGCAAGTATTGGGAATGCAGGATGGCGGATGGCGGAGGCGAGGTAAGGACCGGACGCAAGTT

High #4
WT1-CTGCGGAAGGGCGAGAGCAAGTATTGGGAATGCAGGATGGCGGAGGCGAGGTAAGGACCGGACGCAAATT
WT2-CTGCGGAAGGGCGAGAGCAAGTATTGGGAATGCAGGATGGCGGAGGCGAGGTAAGGACCGGACGCAAGTT
Dl1-CTGCGGAAGGGCGAGAGCAAGTATTGGGAATGCAGGAT------GGCGAGGTAAGGACCGGACGCAAATT
Dl2-CTGCGGAAGGGCGAGAGCAAGTATTGGGAATGCAG-----CGGAGGCGAGGTAAGGACCGGACGCAAATT
Dl3-CTGCGGAAGGGCGAGAGCAAGTATTGGGAATGCAGGATG-CGGAGGCGAGGTAAGGACCGGACGCAAATT

High #9
WT1-CTGCGGAAGGGCGAGAGCAAGTATTGGGAATGCAGGATGGCGGAGGCGAGGTAAGGACCGGACGCAAGTT
Dl1-CTGCGGAAGGGCGAGAGCAAGTATTGGGAATGCAGGA-------GGCGAGGTAAGGACCGGACGCAAGTT
Dl2-CTGCGGAAGGGCGAGAGCAAGTATTGGGAGTGCAGGA-------GGCGAGGTAAGGACCGGACGCAAGTT

High #10
Dl1-CTGCGGAAGGGCGAGAGCAAGTATTGGGAATGCAGGATGG---AGGCGAGGTAAGGACCGGACGCAAATT
Dl2-CTGCGGAAGGGCGAGAGCAAGTATTGGGAATGCAGGA--GCGGAGGCGAGGTAAGGACCGGACGCAAATT

High #11
WT1-CTGCGGAAGGGCGAGAGCAAGTATTGGGAATGCAGGATGGCGGAGGCGAGGTAAGGACCGGACGCAAGTT
Dl1-CTGCGGAAGGGCGAGAGCAAGTATTGGGAATGCAGGATGG---AGGCGAGGTAAGGACCGGACGCAAGTT
In1-CTGCGGAAGGGCGAGAGCAAGTATTGGGAATGCAGGATGGCGCGGAGGCGAGGTAAGGACCGGACGCAAGTT

High #13
Dl1-CTGCGGAAGGGCGAGAGCACGTATTGGGAATGCAGGATGG---AGGCGAGGTAAGGACCGGACGCAAATT
Dl2-CTGCGGAAGGGCGAGAGCAAGTACTGGGAATGCAGGATGG---AGGCGAGGTAAGGACCGGACGCAAATT
Dl3-CTGCGGAAGGGCGAGAGCAAGTATTGGGAATGCAGGATGG---AGGCGAGGTAAGGACCGGACGCAAATT
```

Figure S5 : Off-target mutations analysis.

A. Predicted off-target sites of the sgRNA1 target sequence displaying three mismatches sorted according to their risk score determined at the Zhang portal (http://crispr.mit.edu/). OT1-3 were selected for further study and we verified that no SNPs within OT1-3 sequences were present in (C57BL/6xSJL/J) F1 female and CD1-IGS males. B. Agarose gel electrophoresis analysis of OT3intF-OT3intR PCR products (228bp) of one non-injected embryo (Ctrl) and thirteen embryos (lanes 1 to 13) microinjected into the pronucleus with either low or high Cas9 mRNA/sgRNA1 concentration. Mutant embryos are labelled in red. Black arrowheads point to non-specific bands. Blue arrows point to indels. MW: 100 bp molecular weight marker. C. Sequences of sub-cloned OT3 alleles present in some of the mutant embryos. The off-target sequence is highlighted in gray and its PAM is indicated in bold. Insertions and deletions are labeled in red. A SNP present in genitors is labeled in blue.

site of injection	Cas9/sgRNA (ng/µl)	ssODN matrix (ng/µl)	Nb exp.	Nb embryos*/Nb injected (%)	Nb analyzed	KI (%)	Indels** (%)	WT*** (%)	>2 alleles (%)
Pronucleus	5/2.5	20	2	93/124 (75)	92	31[a] (34)	67 (73)	21 (23)	51 (56)
Pronucleus	100/50	20	3	79/130 (61)	75	40[b] (53)	60 (80)	8 (11)	46 (61)
Cytosol	5/2.5	20	1	59/99 (60)	56	16 (29)	48 (82)	9 (16)	35 (63)
Cytosol	100/50	20	1	31/120 (51)	59	25[c] (42)	45 (76)	6 (10)	27 (46)

Table S1 : Summary of CRISPR/Cas9-mediated *Sox2* mutations obtained after mouse zygotes microinjection

Cas9 mRNA, sgRNA SOX2 and ssODN repair matrix were injected into the cytoplasm or the male pronucleus at 20 ng/µl. Embryos surviving the injection were cultured and embryos that developed up to 8-cell stage and beyond were analysed by PCR and migration of the amplification products on agarose gels. As for the *Notchless* locus, KI efficiency did not depend on the site of injection and increased significantly when more Cas9 mRNA and sgRNA were delivered in the cytosol ($p<0.05$) as well as in the pronucleus ($p<0.01$).

* : 8-cell stage to early blastocyst ; ** : because indels ≤5 bp could be missed by gel electrophoresis analysis, the number of indels is likely underestimated ; *** : WT corresponds to embryos for which no edited alleles was detected.

[a] : including one homozygous KI embryos. [b] : including six homozygous KI embryos. [c] : including five homozygous KI embryo.

sgRNA/ Cas9 variant	HDR matrix (homol. arm)	Cas9/sgRNA (ng/µl)	HDR matrix (ng/µl)	No. KI analyzed	Precise (%)	Imprecise (%)	Both (%)
sgRNA1/ wild-type	ssODN	5/2.5	20	10	6 (60)	4 (40)	0 (0)
		100/50	20	10	8 (80)	1 (10)	1 (10)
	plasmid (500 bp)	100/50	40	6	5 (83)	1 (17)	0 (0)
sgRNA2+sgRNA3 /nickase	ssODN	100/50[†]	20	9	5 (56)	2 (22)	2 (22)
	plasmid (500 bp)	100/50[†]	40	2	1 (50)	1 (50)	0 (0)

Table S2 : Frequency of imprecise KI events

Fidelity of KI events detected in embryos from Table 1 was verified by sequencing. Some embryos displayed only the expected KI allele (precise), some displayed only one or several imprecise alleles (imprecise), and some displayed both the expected allele and an imprecise allele (both). See supplementary figure S3 and S4 for details.

[†] : 50 ng/µl of each sgRNA

Usage	Primer name	Primer sequence
Genotyping	ExtF	CACACGGGTCCCTCTGGCCTTCT
	ExtR	CCTGCCCGCCTAGTCTCTACCTCC
	NleF	GATTTAACGCTCCGTCTTTCC
	NleR	CCTCATCCTGGAACTGTACGA
	HAF	TGAAAAGAAGGCGCGAAG
	HAR	TAATCTGGAACATCATATGGGTACC
	GFPF	GACGAGCTGTACAAGGGAGT
	GFPR	AGTCGGCTCAATCCCAACT
	OT1ExtF	GAGTTAATACTCTGAAGGGT
	OT1ExtR	GGTAAGGAAAGCTGCCATAG
	OT1intF	GGTGATTCTAGTCTGGGTACTT
	OT1intR	GGACTCTGTCCATTATTGGAG
	OT3ExtF	ATTTGAAACTTGGCGGCCC
	OT3ExtR	GGCAAATTCCAAACTCTCCCA
	OT3intF	TCTGAGAAGGAATCGGGTGC
	OT3intR	CCTCCTCGATCCTCCCCT
	Sox2ExtF	AGCTCGCAGACCTACATGAA
	Sox2ExtR	CCCTCAGGTTTTCTCTGTACAA
	Sox2F	GATCAGCATGTACCTCCCCG
	Sox2R	CGTTTCGCTGCGGAGATTTT
Sequencing	WTF1	TACTGTGAAAAGAAGGCGCG
	WTF2	AGAGGATCATGGAGTTCAAGG
	HAF1	ggacgcaggatggGGTACCCA
	HAR2	cgCAGCATAATCTGGAACATC
	GFPF2	TCCGATATTCCCAGCATCCC
	GFPR2	CTGTCCAGGGATGCCATCTC
	seqHAF2	TCCGAGAGCGGCTTGACTCCT
	seqGFPF3	ACGGCCACAAGTTCAGCGTGT
	seqGFPF4	CCTGGTGAACCGCATCGAGCT
	seqOT12	CTTAAGGAGAAAATAGACAGT
	seqOT3	TTAGGAGCACGGGGTGACGAT
	BSB1	AAGGCGATTAAGTTGGGT
	BSB2	GGCTCGTATGTTGTGTGG
Cas9 mRNA synthesis	wtCas9F	gtaatacgactcactatagggagaatgGACTATAAGGACCACGAC
	wtCas9R	GCGAGCTCTAGGAATTCTTAC
	Cas9nF	gtaatacgactcactatagggagaatgTACCCATACGATGTTCCAGATTACGC
	Cas9nR	GCGAGCTCTAGGAATTCTTAG
sgRNA synthesis	sgRNA1F	caccGTGGGGACGCAGGATGGCGG
	sgRNA1R	aaacCCGCCATCCTGCGTCCCCAC
	sgRNA2F	caccCCCCACGTGGAGGAGAACCG
	sgRNA2R	aaacCGGTTCTCCTCCACGTGGGG
	sgRNA3F	caccTGGTGGTAGGTGTACGCGCA
	sgRNA3R	aaacTGCGCGTACACCTACCACCA
	sgSox2F	caccGTGCCCCTGTCGCACATGTGA
	sgSox2R	aaacTCACATGTGCGACAGGGGCAC
	T7-sgRNA1	gtaatacgactcactatagggGTGGGGACGCAGGATGGCGG
	T7-sgRNA2	gtaatacgactcactatagggCCCCACGTGGAGGAGAACCG
	T7-sgRNA3	gtaatacgactcactatagggTGGTGGTAGGTGTACGCGCA
	T7-sgSox2	gtaatacgactcactatagggGTGCCCCTGTCGCACATGTGA
	allsgRNAR	AAAAGCACCGACTCGGTGCC
Reparation matrix synthesis	NleGFP250F	CTCCTGCCTCTGTGAGCTAGG
	NleGFP250R	GCGCGTTGCACACCAGCTGCA
	NleGFP60F	TAACGCTCCGTCTTTCCGCCT
	NleGFP60R	CCCCACGCGGAGACCCGGCAT

Nle-HA ssODN	ACATGGATTTAACGCTCCGTCTTTCCGCCTCGGTTCTCCTCCACGTGGGGACGCAGGATG GGGTACCCATATGATGTTCCAGATTATGCTGCGGCGGCGGTGGTGGTAGGTGTACGCGC ACGGGGCGGGGATGCCGGGTCTCCGCGTGGG
Sox2-HA ssODN	TACCAGAGCGGCCCGGTGCCCGGCACGGCCATTAACGGCACACTGCCCCTGTCGCACAT GTACCCATATGATGTTCCAGATTATGCTTGAGGGCTGGACTGCGAACTGGAGAAGGGGA GAGATTTTCAAAGAGATACAAGGGAATTG

Table S3 : Oligonucleotides used in this study
